# The Stylohyoid Complex: An Update on Its Embryology, Comparative Anatomy and Human Variations

**DOI:** 10.3390/biology14111500

**Published:** 2025-10-27

**Authors:** Maria Piagkou, George Triantafyllou

**Affiliations:** 1 Department of Anatomy, Faculty of Health Sciences, School of Medicine, National and Kapodistrian University of Athens, 11527 Athens, Greece; georgerose406@gmail.com; 2 “VARIANTIS” Research Laboratory, Department of Clinical Anatomy, Masovian Academy in Płock, 09400 Płock, Poland

**Keywords:** styloid process, stylohyoid chain, developmental anatomy, comparative anatomy, variation, carotid arteries, clinical anatomy

## Abstract

**Simple Summary:**

The stylohyoid complex, composed of the styloid process, stylohyoid ligament, and lesser horn of the hyoid, originates from Reichert’s cartilage of the second pharyngeal arch. Though vestigial in humans, it occupies a key position in the parapharyngeal space, interfacing with the internal and external carotid arteries, internal jugular vein, and lower cranial nerves. Embryological and comparative studies reveal its evolutionary reduction from an ossicular chain to a slender process, enabling vocal tract flexibility. Human variations in length, angulation, and ossification underpin Eagle’s syndrome and vascular complications, including carotid dissection and jugular compression, making this complex clinically significant.

**Abstract:**

The stylohyoid complex (SHC), comprising the styloid process (SP), stylohyoid ligament, and lesser horn of the hyoid bone, arises from Reichert’s cartilage and plays a central role in head and neck organization. Although anatomically small, it occupies a strategic position in the parapharyngeal space, linking neural, vascular, and visceral compartments. This review integrates embryological, comparative, anatomical, and clinical perspectives to provide an updated synthesis of SHC morphology and significance. Developmental studies highlight the early segmentation of Reichert’s cartilage, its transient relationships with the otic capsule, facial canal, and carotid arteries, and its role in shaping muscular and fascial compartments. Comparative anatomy demonstrates the evolutionary transition from a continuous ossicular chain to a vestigial human structure, reflecting a trade-off between rigidity and vocal tract flexibility. In humans, the SHC exhibits marked variability in length, angulation, segmentation, and ligamentous ossification, which directly influence its spatial relationships with the internal and external carotid arteries, the internal jugular vein, and the lower cranial nerves. These variations underpin the clinical spectrum of Eagle’s syndrome and vascular complications, including carotid artery dissection and jugular compression syndromes. Recognition of these embryological origins, evolutionary trajectories, and anatomical variants is essential for accurate diagnosis, imaging interpretation, and surgical planning. As both an embryological remnant and a clinical landmark, the SHC bridges fundamental anatomy with practical implications for imaging, diagnosis, and surgery.

## 1. Introduction

Developmental and evolutionary biology provide valuable insights into key questions of human anatomy. Comparative and embryological studies have long supported the understanding of both typical and variant human morphology [1,2]. Among the most intriguing structures reflecting this complexity is the skull, which has been extensively examined through histological, embryological series, and comparative morphological analyses [3,4]. Within this context, the styloid process (SP)—a slender bony projection of the temporal bone—stands out as a structure of considerable clinical and anatomical interest. In this review, we analyze its developmental and evolutionary background and discuss its clinical relevance.

The head and neck region contains essential neurovascular structures intricately related to bony landmarks and surrounding soft tissues. This region is one of the most anatomically complex areas of the body, containing the special sense organs, the upper aerodigestive tract, and an elaborate neurovascular network [5]. Anatomically, the skull base forms the structural foundation, transmitting cranial nerves (CNs) and blood vessels through multiple foramina, while the visceral compartment contains the pharynx, larynx, trachea, and esophagus [5]. The vascular system is composed of the paired common carotid arteries (CCAs), which bifurcate into the internal carotid arteries (ICAs) and the external carotid arteries (ECAs), along with the internal jugular veins (IJVs). These are enclosed by the carotid sheath together with the vagus nerve (X) and the sympathetic chain [5]. All these elements occupy the parapharyngeal space, a deep cervical compartment extending from the skull base to the hyoid bone (HB). Traditionally divided into prestyloid and retrostyloid compartments, its exact boundaries remain debated [6]. More recent syntheses propose the styloid diaphragm as the most reliable divider, while the ten-sor-vascular-styloid fascia further partitions the prestyloid compartment into parotid, paramasticatory, and paratonsillar regions [6]. These compartments are defined by muscular planes responsible for mastication, swallowing, phonation, and head movements [5]. Within this intricate organization, the SP and its muscular derivatives—styloglossus, stylopharyngeus, and stylohyoid—serve as essential topographic landmarks. By traversing the parapharyngeal space, they demarcate neural, vascular, and visceral compartments.

The SP arises from the temporal bone, a paired cranial element forming part of the lateral skull base. The temporal bone contributes to both the middle and posterior cranial fossae and is subdivided into four distinct parts: the squamous, tympanic, mastoid, and petrous parts [5]. The squamous part forms the lateral cranial vault and zygomatic arch, the tympanic part creates the external auditory canal boundaries, and the mastoid part contains the mastoid air cells. The petrous portion, the most complex, houses the cochlea, vestibule, semicircular canals, and the internal acoustic meatus, accommodating the auditory and vestibular organs as well as the intrapetrous segment of the facial nerve (FN) [7,8]. Modern imaging and anatomical studies have underscored remarkable variability in the temporal bone [9,10]. Among these variations, the SP itself shows notable morphological diversity.

The stylohyoid complex (SHC) comprises the SP, the stylohyoid ligament (SHL), and the lesser horn (LH) of the HB. The SP is a slender, cylindrical projection emerging from the petrous temporal bone, posterior to the tympanic plate and vaginal process, which partially obscures its origin [5,11]. Its typical length ranges from 2.5 to 3.0 cm [12,13], though reported measurements vary from 1.5 to 4.8 cm. Natsis et al. [11] classified SPs shorter than 18 mm as “short,” 18–33 mm as “typical,” and greater than 33 mm as “elongated,” with elongation observed in approximately 28% of their Greek sample.

Functionally, the SP anchors three paired muscles (styloglossus, stylohyoid, stylo-pharyngeus) and two paired ligaments (stylohyoid and stylomandibular) [5]. These attachments allow coordinated movement of the mandible, HB, tongue, and pharynx, underscoring the structural and functional role of the SHC.

Anatomically, the SP lies between the carotid arteries (CAs), making its vascular relationships clinically relevant. The CCA typically bifurcates at the level of C3–C4 or the upper thyroid cartilage into the ICA and ECA. While the ICA usually ascends in a straight, unbranched course toward the skull base, the ECA runs anteriorly and laterally, branching to supply the face, scalp, pharynx, and dura mater [5,14]. Considerable variability, however, has been documented. The ICA may exhibit tortuosity, kinking, or coiling in up to 26% of individuals [14], which can bring the artery into proximity with the pharyngeal wall or tonsillar fossa, posing risks during pharyngeal surgery and contributing to cerebrovascular symptoms. The ECA also demonstrates variability in branching patterns [15,16] and topographic course [17,18]. Recent computed tomography angiography (CTA) studies have shown retrostyloid displacement of the ECA in ~9% of cases and distinctive retromandibular loops in up to 45%, variants that directly alter its spatial relationship with the SP, mandible, and parapharyngeal space [17,18].

By integrating both developmental and evolutionary perspectives, similar to previous reviews [1], we will present an update of the SP embryology, comparative anatomy and subsequently its clinical significance in humans.

## 2. Embryological Development of the Stylohyoid Complex

The SHC, comprising the SP, SHL, and the LH of the HB, derives from the second pharyngeal arch cartilage (Reichert’s cartilage, RC). Classical and modern embryological studies have clarified the morphogenesis and segmentation of this process. However, it also interacts closely with neighboring structures (FN, otic capsule, cranial vessels) and undergoes partial regression that defines the adult arrangement.

### 2.1. Early Segmentation of the Reichert’s Cartilage

RC appears as a mesenchymal condensation along the second arch by Carnegie stage 17 (6 weeks of development), lying between the developing pharynx and the facial nerve [19]. By stage 18, chondrogenesis begins in the cranial portion, which will form the SP, while the caudal portion remains in a precartilaginous phase and later contributes to the LH of the HB [19].

Embryological series show that RC is never a single continuous bar; rather, it forms in two independent segments:

- A cranial or styloid segment, continuous with the otic capsule, curving in a hook-like fashion, and closely related to the ECA and cranial nerves IX and X [20];
- A caudal or hyoid segment, which develops into the lesser horn of the hyoid [20].

Between these, a mesenchymal bridge persists until 7–8 weeks but usually regresses by 10 weeks, leaving no true cartilaginous continuity [19].

### 2.2. Relationship with Surrounding Structures-Otic Capsule, Stapes and Nerves

The cranial (styloid) segment remains attached to the otic capsule, contributing to the vertical portion of the facial canal (FC) [20]. Histological reconstructions from fetal series (19–34 weeks) revealed that the RC transiently forms part of the tympanic wall before being replaced by membranous bone as the FC and tympanic cavity expand [21]. The angulated inferior end of the styloid segment is consistently seen near the ECA and pharyngeal wall, anticipating the close neurovascular relationships of the adult SP [20].

Therefore, neurovascular relationships are established early as follows:

- The FN runs lateral to the styloid segment [22],
- The glossopharyngeal, vagus, accessory, and hypoglossal nerves descend near RC, separated by the ICA and IJV [22],
- The styloid segment angulated inferior end lies beside the ECA and cranial nerves IX–X [19].

### 2.3. Ossification and Persistency Processes

In near-term fetuses (25–40 weeks), endochondral ossification of the cranial segment produces the definitive SP. The caudal segment ossifies as the lesser horn of the hyoid. The intervening mesenchyme does not usually chondrify, and its regression explains the presence of the SHL in adults [19]. Li et al. [23] showed that, in near-term fetuses (25–40 weeks), the cranial part of RC often exhibits branched or T-shaped morphology, with one projection toward the tympanic cavity and another toward the FC [23]. Variations in branching and fusion with the petrosa likely reflect mechanical influences from the developing petrosal and tympanic bones, and they may account for the diversity of styloid root morphologies observed in adults [23].

### 2.4. Associated Muscular, Fasciae and Connective Derivatives

RC also provides the origins of the styloid muscles (styloglossus, stylohyoid, and sty-lopharyngeus). Histological studies confirm that these muscles first arise directly from RC, not the temporal bone [24]. Variations in fetal origins (e.g., accessory fascicles from the mandibular angle) highlight RC’s transitional role as a scaffold for both bone and muscle [24].

In near-term fetuses (25–40 weeks), the SP and its muscles help organize the fascial compartments of the parapharyngeal space. The carotid sheath, superior cervical sympathetic ganglion, and lower CNs are enclosed within fascial layers influenced by styloid derivatives [25]. This embryonic arrangement explains the adult prestyloid versus retrostyloid division, with the styloid muscles forming the anatomical boundary. However, recent evidence has questioned these traditional definitions [6].

### 2.5. Developmental and Evolutionary Implications

The disconnection of cranial and caudal segments of RC during development may have facilitated the descent of the HB in humans [19]. This developmental arrangement also provides a basis for the common variations in the SHC, such as elongation or ossification of the SHL, which are implicated in Eagle’s syndrome, and it will be discussed in the following sections (Table 1).

## 3. Comparative Anatomy of the Stylohyoid Complex

The SHC is one of the most variable components of the mammalian head and neck system. While humans retain only a reduced SP connected to the HB by a ligament, other vertebrates preserve a more elaborate chain of ossicles, reflecting adaptations in feeding, respiration, and communication [26].

### 3.1. General Mammalian Pattern

In mammals, the hyoid apparatus typically consists of a suspensory portion (tympanohyal, stylohyal, epihyal, ceratohyal) linking the skull to a basal portion (basihyal and thyrohyals) that anchors the tongue and larynx. This arrangement is relatively conserved but highly variable in detail [27].

### 3.2. Primates

Hilloowala [28] documented striking differences among primates. A distinct SP was consistently present in baboons, rudimentary in macaques, and absent in smaller primates such as marmosets. Howler monkeys exhibited a disproportionately enlarged basihyal, reflecting adaptation for loud calls. These findings link SHC variation with both vocalization capacity and craniofacial scaling [28].

### 3.3. Rodents and Lagomorphs

Sprague [29] showed that in woodrats (Neotoma), the tympanohyal develops as a separate element but later fuses with the mastoid, while the stylohyal persists as a styliform rod. Anapol [30] found that in rabbits, the hyoid apparatus lacks a firm bony connection to the skull and is instead ligamentously suspended, providing stability for tongue movements during chewing and swallowing. These models parallel the human condition, where ligamentous reduction increases mobility.

### 3.4. Carnivores

The carnivore hyoid typically consists of the stylohyal, epihyal, ceratohyal, and basihyal, which are suspended by a tympanohyal. Takada et al. [27] noted that in canids, ursids, and viverrids, the tympanohyal is a discrete ossicle within the stylomastoid foramen, whereas in mustelids it is obscured by the tympanic bulla. Weissengruber et al. [31] described felid variation in “roaring cats” (lion, tiger, jaguar), where the epihyal is ligamentous, allowing for laryngeal descent and elongation of the vocal tract. In contrast, cheetahs and domestic cats have an ossified epihyal, enabling purring but not roaring. Flores et al. [32] further demonstrated that dire wolves had more robust hyoids than modern gray wolves, likely an adaptation for lower-frequency vocalizations.

### 3.5. Ungulates

Hartl et al. [33] demonstrated that horses possess a highly specialized temporohyoid joint, where the stylohyoid is massive and articulates with the petrous temporal bone via synchondrosis. In ruminants, the joint is synovial, while in pigs, the epihyal is replaced by a ligament [33]. These variations correspond to grazing mechanics and airway support, reflecting distinct tongue biomechanics across ungulates.

### 3.6. Proboscideans

In elephants and their extinct relatives, Shoshani and Marchang [34] reported that the epihyal and ceratohyal are absent, leaving a gap between the stylohyal and the basihyal–thyrohyal unit. The stylohyal is massive and bifurcated (Y-shaped), providing broad muscle attachment and stabilizing the hyoid deep in the neck. This supports trunk–tongue coordination, infrasonic vocalizations, and pharyngeal water reservoirs [34].

### 3.7. Cetaceans

Reidenberg and Laitman [35] demonstrated that odontocete whales possess a fully ossified, flattened hyoid apparatus that is enlarged and tilted compared to terrestrial mammals. This configuration provides a strong anchor for tongue and pharyngeal muscles used in suction feeding and echolocation sound production. Unlike humans, cetaceans preserve continuous ossicular articulations [35].

### 3.8. Xenarthrans and Fossil Glyptodonts

Pérez et al. [36] and Zamorano et al. [37] described xenarthrans (sloths, anteaters, armadillos) as having unusual fusion patterns. In glyptodonts, the stylohyal, epihyal, and ceratohyal fuse into a single “sigmohyal,” while the basihyal and thyrohyals fuse into a “V-bone.” Panochthus exhibited more gracile, elongated elements than Glyptodon, with stronger musculature enabling greater tongue mobility and dietary flexibility. In contrast, giant sloths had more rigid hyoids, suited for intraoral food processing [37].

### 3.9. Functional Trends

From these comparisons, two broad evolutionary trajectories emerge:

- Ossified continuity (cetaceans, ungulates, many carnivores, elephants): supports powerful and specialized feeding or vocal functions (suction feeding, grazing, roaring, infrasonics).
- Reduction and ligamentous suspension (primates, rodents, lagomorphs, humans): enhances cranial mobility, facilitates hyoid descent, and in humans, underpins the evolution of speech.

Thus, the human SP is best understood as a vestigial remnant of a once extensive chain, whose evolutionary reduction reflects a tradeoff favoring vocal tract flexibility over structural rigidity (Table 2).

## 4. Human Variations and Relationships with Neurovascular Structures

Having delineated the evolutionary diminution of the SHC throughout mammals, we now address its variability in humans, wherein anatomical polymorphisms bear direct clinical significance. Human SHC is highly polymorphic. While the SP is traditionally described as a slender projection averaging 2.5–3.0 cm in length, modern anatomical and radiological studies demonstrate a far broader spectrum of morphologies. Documented variations include differences in length, angulation, segmentation, duplication, and ossification of the stylohyoid ligament, as well as diverse patterns of articulation. These morphological variants significantly alter the SHC’s spatial relationships with adjacent neurovascular structures and underpin its clinical relevance.

### 4.1. Length Variability

The definition of an elongated SP has historically varied. Eagle [13] set the normal range at 25–30 mm, but large radiographic series show broader variation. Jung et al. [38], analyzing 1000 panoramic radiographs, proposed a threshold of >45 mm to define elongation, since values above 30 mm were common in normal patients [38]. Monsour and Young [39] similarly reported that approximately 21% of SPs exceeded 40 mm, with steady growth into the third decade and a secondary increase after the age of 60 years [39]. Stafne et al. [40] documented lengths from 5 to 50 mm in roentgenographic and anatomical studies [40]. Nevertheless, Natsis et al. [11] recorded the 33 mm threshold for an elongated SP based on the confidence intervals of their population.

More recently, Triantafyllou et al. [41], in a systematic review of 136,010 heminecks, calculated a pooled mean length of 28.91 mm and an elongation prevalence of 25.03%, confirming that elongation is not a rare anatomical variant. Nevertheless, the same meta-analysis did not identify any statistically significant difference between sexes [41]. This was also confirmed by isolated studies that did not observe sexual dimorphism in their sample [38,39,40]. The age effect on the SP elongation was not examined from the meta-analysis due to a lack of evidence [41]. Monsour and Young [39] reported that SP length increases progressively into the third decade, followed by a secondary increase after the age of 60 years. Similarly, Jung et al. [38] demonstrated that the upper percentile of SP length increases with age, especially in males over 35 years. Thus, SP elongation may have both congenital and acquired components, with a tendency toward progressive ossification in later decades.

**Figure 1:**
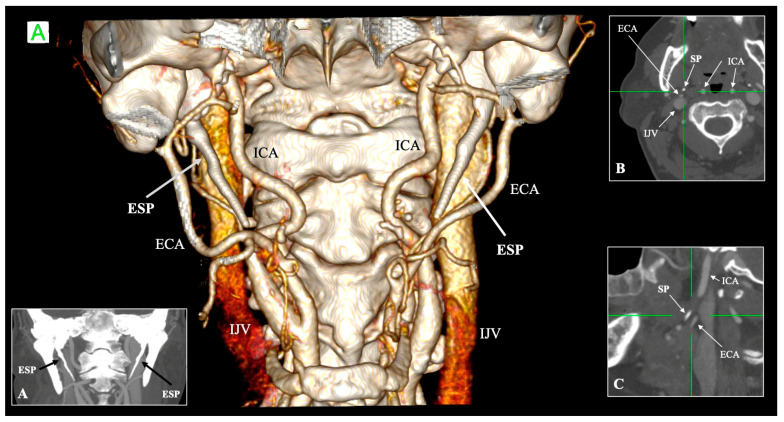
Three-dimensional, coronal (**A**), axial (**B**) and sagittal (**C**) reconstructions showing bilateral elongated styloid process (ESP) with a close relationship to the external and internal carotid artery (ECA and ICA). Note that the ECA has a retrostyloid course—posterior to the styloid process—as depicted in the sagittal plane (**C**). IJV—internal jugular vein, SP—styloid process, A—anterior.

The meta-analysis confirmed that CT is the most reliable method by presenting the highest pooled prevalence of elongated SP [41] (Figure 1).

### 4.2. Angulation and Orientation

Orientation is equally variable. Andrei et al. [42], using cone-beam CT, measured both sagittal and transverse angulations and found correlations between them, noting that elongated SPs displayed significantly different sagittal angles compared to normal ones [42]. Onbas et al. [43] described transverse angles of 55–90.5° and sagittal angles of 76–110° on multidetector CT [43]. Such deviations alter the SP’s relationship with vascular and neural structures.

### 4.3. Morphological Variations

Morphological diversity of the SP includes segmentation, duplication, absence, and pseudoarticulations. Stafne et al. [40] described complete separation of the tympanohyal, stylohyal, and epihyal elements, along with curved processes and bulbous enlargements at fusion points [40]. Monsour and Young [39] reported segmentation in 35% of cases and rare duplication [39]. Onbas et al. [43] confirmed duplication in 3.1% of individuals and absence in 2.5% [43]. Ramadan et al. [44] emphasized that CT is the most reliable modality for identifying these variants, as cadaveric studies often miss thin or incomplete ossifications [44].

### 4.4. Ossification of the Stylohyoid Ligament

**Figure 2:**
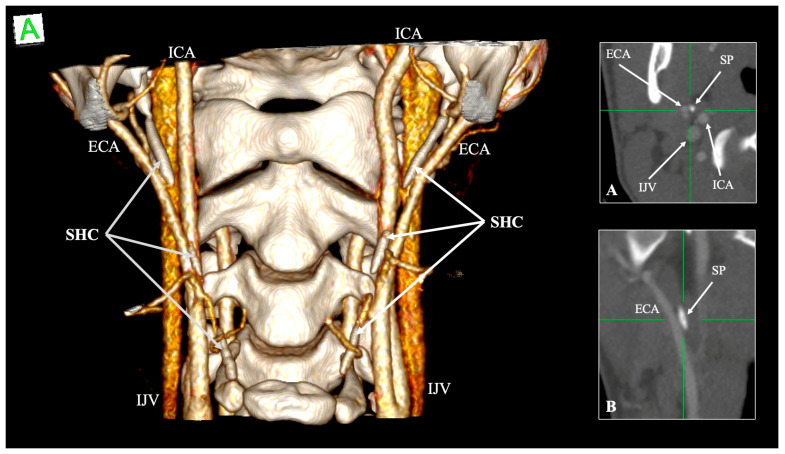
Three-dimensional, axial (**A**) and coronal (**B**) reconstructions showing variable ossification of the stylohoid chain (SHC) with a very close relationship (“in contact”) with the external carotid artery (ECA). ICA—internal carotid artery, IJV—internal jugular vein, SP—styloid process, A—anterior.

Ossification of the SHL, historically termed “ossified stylohyoid chain,” is a frequent finding. Stafne et al. [40] described partial and complete ossification of the epihyal (ceratohyal) element, sometimes bilateral [40]. Monsour and Young [39] found ossification in ~9% of panoramic radiographs [39], while Onbas et al. [43] reported ossification in 13.4% of their CT cohort [43]. Ramadan et al. [44] categorized ossification into complete, partial, and segmented, stressing its frequent incidental discovery during imaging for unrelated conditions [44]. In the recent meta-analysis, Triantafyllou et al. [41] quantified pseudoarticulated and segmented SHC ossifications at pooled prevalences of 4.39% and 3.89%, respectively [41] (Figure 2).

### 4.5. Relationship with Neurovascular Structures

The SP occupies the retro- and parapharyngeal space, where it is interposed between the ICA, ECA, and IJV. Modern angiographic and morphometric studies confirm that its variable length, angulation, and ossification patterns substantially influence these vascular relationships.

**Figure 3:**
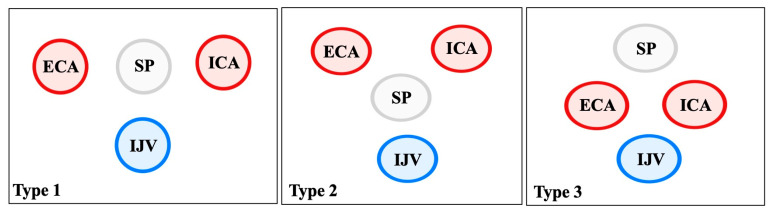
The three topographical patterns of the styloid process (SP) tip delineated through Triantafyllou et al. [48] study. In Type 1, the most common configuration, the SP lies between the ICA and ECA, with the IJV posteriorly positioned, forming a reliable surgical landmark. In Type 2, the SP is located anterior to the IJV, separating it from the CAs; this arrangement predisposes to venous compression, particularly in cases of elongation or medial deviation, and underlies the jugular variant of Eagle’s syndrome. In Type 3, the SP is situated superiorly, while the ICA and ECA course closely together anterior to the IJV, creating a narrow retrostyloid corridor and increasing the risk of arterial contact or dissection. Recognition of these spatial variants is essential for both radiological interpretation and surgical planning.

The ICA is the structure most frequently implicated with SP variants. In their innovative study, Raser et al. [45] showed that longer SP are independently associated with cervical ICA dissection, with patients in the highest quartile of length having a fourfold higher risk [45]. Amorim et al. [46] corroborated these findings in a larger multicenter cohort: elongated SP ipsilateral to dissection averaged 35.8 mm compared to 30 mm in controls, and the SP–ICA distance was significantly reduced [46]. Renard et al. [47] further emphasized that closer anatomical relationship, rather than elongation alone, is the critical factor: ICA–SP distances were significantly shorter on the side of dissection than in controls, and direct bony–arterial contact was also more prevalent [47]. Triantafyllou et al. [48] quantified these topographies, reporting mean SP–ICA distances of 6.16 mm at the tip and 4.72 mm at the point of closest approach. Both elongation and ossified SHC segments significantly shortened this distance [48] (Figure 3). In a follow-up study, Triantafyllou et al. [49] demonstrated that angulation alone (sagittal/coronal) did not significantly influence ICA proximity when elongation was excluded, confirming that length is the primary determinant [49] (Table 3).

Traditionally regarded as lateral to the SP, the ECA may course posteriorly (retrostyloid) in a minority of cases (Figure 1). Calotă et al. [50] found retrostyloid ECA in 11.9% of 320 heminecks studied, often coexisting with elongated SP [50]. Karangeli et al. [17] similarly reported a 9% prevalence of retrostyloid ECA, unaffected by SP elongation or ossification [17]. Triantafyllou et al. [48] documented mean SP–ECA distances of 5.45 mm at the tip and 3.96 mm at the narrowest point [48], underlining the limited surgical corridor between the process and artery (Table 3).

Although less frequently discussed, the IJV lies posterolateral to the SP and may be compressed in cases of marked elongation or abnormal angulation. Modern imaging studies [48] show the IJV within the same narrow retrostyloid compartment, where the SP can reduce the venous space depending on head and neck position [48] (Table 3).

Beyond vascular relations, the SP is also intimately associated with several CNs in the parapharyngeal space. Laterally, the FN exits the stylomastoid foramen just posterior to the SP base (Table 3). Medially, the glossopharyngeal (CN IX), vagus (CN X), accessory (CN XI), and hypoglossal (CN XII) nerves descend adjacent to the ICA and IJV [43,48,49]. Elongation or medial angulation of the SP narrows the interval between the process and these nerves, predisposing particularly CN IX–XII to compression in the retrostyloid compartment. Katori et al. [25] emphasized that SP and its muscular derivatives establish a fascial boundary dividing the parapharyngeal space into prestyloid and retrostyloid compartments, the latter enclosing the lower CNs within the carotid sheath. These relationships explain the frequent neural involvement observed in cases of marked SP variation.

## 5. Clinical Anatomy of the Stylohyoid Complex

The anatomical diversity of the SHC acquires clinical importance when variations in length, angulation, or ossification alter its relationships with adjacent neurovascular and neural structures. These variations form the anatomical basis of Eagle’s syndrome and its vascular variants. Eagle’s seminal reports [12,13] first established the clinical significance of an elongated SP, distinguishing between the “classic syndrome,” characterized by pharyngeal and otologic pain, often following tonsillectomy, and the “carotid syndrome,” which results from mechanical irritation of the internal or external carotid artery and their associated sympathetic plexuses. These foundational observations laid the groundwork for understanding how diverse SHC morphologies can manifest in a spectrum of clinical symptoms.

Radiological studies have shown elongation (>30 mm) in 4–30% of individuals, though only 4–10% are symptomatic [51,52]. Three main variants are recognized:

- Classic Eagle syndrome: Presents with throat pain, dysphagia, foreign body sensation, otalgia, or pain on head rotation. Symptoms are explained by close relations of the SP with CN V, VII, IX, and X [51]. Often occurs after tonsillectomy, where local scar tissue increases nerve traction [13].
- Carotid (stylocarotid) variant: Involves irritation or compression of the ICA or ECA. Patients may present with hemicranial headache, periorbital pain, transient ischemic attacks, or carotid artery dissection [13,53,54]. Dynamic head rotation can worsen contact, explaining symptom fluctuation.
- Jugular variant (Eagle jugular syndrome): Zamboni et al. [55] described compression of the IJV between the SP and the C1 transverse process, producing intracranial hypertension, papilledema, venous congestion, and even peri-mesencephalic hemorrhage [55]. Headache and orbital symptoms dominate, distinguishing it from the carotid subtype [56].

Case-based radiological studies reinforce these patterns. Rohee-Traoré and Boucher [52] showed a 7.4-cm ossified SHC fused to the hyoid horn, palpable in the tonsillar fossa, explaining odynophagia and retromolar pain [52].

Diagnosis integrates clinical and imaging criteria:

- Palpation of the tonsillar fossa reproducing pain remains pathognomonic [57].
- Local anesthetic infiltration provides temporary relief and diagnostic confirmation [57].
- CT with 3D reconstruction is the gold standard for measuring length, angulation, and ossification [48,49,52].
- Angio-CT and Doppler ultrasound are critical for vascular subtypes, especially ICA dissection and IJV compression [54,55,56].
- Dynamic imaging is increasingly used to capture positional compression [58,59].

Conservative treatment (analgesics, NSAIDs, corticosteroid infiltration) can alleviate pain in mild cases [52], but most symptomatic patients eventually require surgery. Intraoral styloidectomy, which was first described by Eagle [12,13], allows direct access but with limited visualization and a higher risk of neurovascular injury [13]. Transcervical styloidectomy offers wider exposure of ICA, IJV, and cranial nerves, reducing operative risk [54]. Minimally invasive approaches are also possible with transoral robotic surgery, with improved visualization and precision and favorable outcomes [58]. The reported outcomes achieved higher success rates than conservative therapy. Hassani et al. [60] reported improvement in ~98% of surgical patients compared to ~66% managed medically [60]. In vascular subtypes, styloidectomy may prevent catastrophic complications such as dissection or stroke [59].

Although Eagle’s syndrome is classically described along with SP elongation, recent radiological and clinical evidence shows that elongation or ossification of the stylohyoid complex may contribute to a broader spectrum of pathologies [45,61]. Several angiographic and case–control studies have confirmed an association between elongated SP and cervical carotid artery dissection (CCAD). Raser et al. [45] showed that patients in the highest quartile of styloid length had a fourfold increased risk of CCAD [45]. Amorim et al. [46] found a significantly shorter SP–ICA distance in dissection patients, and Renard et al. [47] demonstrated that direct bony–arterial contact was more common ipsilateral to dissections [46,47]. Clinical consequences include transient ischemic attacks, ischemic strokes, Horner’s syndrome, and neck pain [45]. The jugular variant of Eagle syndrome, described by Zamboni et al. [55], results from compression of the IJV between the styloid process and the C1 transverse process. This “jugular nutcracker” phenomenon may cause intracranial hypertension, papilledema, venous congestion, or even subarachnoid hemorrhage [55,56]. Thielen et al. [59] confirmed that dynamic imaging reveals IJV narrowing in head rotation, supporting the need for CT venography in suspected cases [59].

Beyond its clinical significance in Eagle syndrome and head and neck surgery, the SHC also holds substantial relevance in forensic medicine. Anatomical variations or elongation of the SP may predispose individuals to fracture during neck compression events, such as hanging or manual strangulation. Radiological studies have established that elongated or ossified elements of the SHC may mimic traumatic fractures, requiring careful differentiation by forensic [62]. Fractures of the ossified SHC or elongated SP have been reported as indicators of homicidal strangulation, whereas their absence in younger victims may be attributed to higher ligamentous elasticity [62]. Conversely, elderly individuals demonstrate increased risk of fracture due to age-related ossification and ankylosis of the hyoid apparatus [63]. Importantly, in some autopsy cases, an elongated styloid process itself has been implicated as a cause of sudden death via carotid artery impingement or vagally mediated cardiac inhibition, even in the absence of external trauma [62,63].

## 6. Conclusions

The SHC exemplifies how vestigial embryological structures, shaped by evolutionary pressures, may retain profound anatomical and clinical significance. The SHC, derived from RC, exemplifies how embryological remnants can shape adult anatomy and pathology. Its evolutionary reduction from a complete ossicular chain to a vestigial SP with ligamentous suspension enhanced vocal tract flexibility but also introduced structural variability. In humans, marked variation in length, angulation, and ossification alters its relationships with the ICA, ECA, IJV, and cranial nerves. These anatomical differences underpin the spectrum of clinical syndromes first described by Eagle, now extended to include vascular and jugular variants associated with dissection, stroke, and intracranial hypertension. Recognition of these patterns is essential. Although most cases are incidental, symptomatic patients benefit from accurate imaging, dynamic assessment, and, when necessary, surgical resection with modern minimally invasive techniques. Thus, the SHC remains a small but clinically significant structure where embryology, evolution, and anatomy converge.

## Figures and Tables

**Table 1:** Summary of the key developmental stages of the Reichert’s cartilage (RC) and the stylohyoid complex (SHC).

Developmental Stage	Key Features of RC	Relationships with Adjacent Structures	References
Carnegie stage 17 (6 weeks)	RC appears as a mesenchymal condensation in the 2nd pharyngeal arch	Lies between the pharynx and the FN	Rodríguez-Vázquez et al. [19]
Carnegie stage 18 (6–7 weeks)	Cranial segment begins chondrogenesis → future SP- caudal segment remains precartilaginous → future LH of the HB	Close to otic capsule, ECA, CN IX–X	Rodríguez-Vázquez et al. [20]
7–8 weeks	A mesenchymal bridge transiently connects cranial (styloid) and caudal (hyoid) segments	ICA and IJV separate RC from CN IX–XII	Rodríguez-Vázquez et al. [19]; Cho et al. [22]
~10 weeks	Regression of mesenchymal bridge → no continuous cartilage	The FN runs laterally to the styloid segment	Cho et al. [22]
19–34 weeks (fetal series)	The styloid segment forms part of the tympanic wall before being replaced by the membrane bone	The vertical portion of the FC is shaped	Anson et al. [21]
25–40 weeks (near term)	The cranial part shows branched/T-shaped morphology, projecting toward the tympanic cavity and the FC	Fusion with petrosal & tympanic bones influences styloid root morphology	Li et al. [23]
	Cranial segment undergoes endochondral ossification → SP; Caudal segment also undergoes endochondral ossification →LH of the HB	Muscles (styloglossus, stylohyoid, stylopharyngeus) originate from the RC; the styloid fascia divides the parapharyngeal space	Mérida-Velasco et al. [24]; Katori et al. [25]

**Table 2:** Summary of the key comparative anatomy of the stylohyoid complex (SHC).

Taxon/Group	Main SHC Elements	Distinctive Features	Functional Adaptations	References
Mammals (general)	Tympanohyal, stylohyal, epihyal, ceratohyal, basihyal, thyrohyal	Conserved suspensory + basal portions	Tongue and laryngeal support	Takada et al. [27]; Weissengruber et al. [31]
Primates	Reduced SP; variable hyoid ossicles	SP is distinct in baboons, rudimentary in macaques, and absent in small primates	Variation linked to vocalization and craniofacial size	Hilloowala [28]
Rodents/Lagomorphs	Ligamentous suspension of the HB	Tympanohyal fuses to the mastoid; stylohyal slender rod	Stable base for chewing and swallowing; mobility of the tongue	Sprague [29]; Anapol [30]
Carnivores (Felids, Canids, Ursids, Mustelids)	Ossified stylohyal, variable epihyal	Roaring cats: epihyal ligamentous; small cats: epihyal ossified	Roaring vs purring mechanisms; laryngeal descent	Weissengruber et al. [31]; Takada et al. [27]
Ungulates	Massive stylohyal + variable epihyal	Horses: temporohyoid synchondrosis; ruminants: synovial joint; pigs: epihyal replaced by ligament	Supports grazing mechanics and airway stability	Hartl et al. [33]
Proboscideans (Elephants)	Stylohyal Y-shaped; epihyal/ceratohyal absent	Fusion with basihyal-thyrohyal unit; deep neck stabilization	Supports trunk–tongue coordination and infrasonic calls	Shoshani and Marchang [34]
Cetaceans	Fully ossified, flattened hyoid	Enlarged and tilted apparatus	Anchor for suction feeding, echolocation sound production	Reidenberg and Laitman [35]
Xenarthrans/Fossil Glyptodonts	Fusion into sigmohyal and V-bone	Glyptodonts: rigid fusion; sloths: elongated, gracile elements	Dietary specialization and tongue mobility	Pérez et al. [36]; Zamorano et al. [37]

**Table 3:** Summary of the key neurovascular relationships of the stylohyoid complex (SHC).

Structure	Typical Relationship with SP/SHC	Variant/Altered Relationship	Clinical Significance	References
Internal carotid artery (ICA)	Medial to SP, ascends branchless to the skull base	Tortuosity, kinking, coiling; reduced ICA–SP distance; direct bony contact	Risk of dissection, ischemic stroke, and hemorrhage during pharyngeal surgery	Paulsen et al. [14]; Renard et al. [47]; Raser et al. [45]; Amorim et al. [46]
External carotid artery (ECA)	Anterolateral to SP; gives facial and pharyngeal branches	Retro-styloid course (~9–12%)	Altered surgical corridor; potential compression or irritation	Karangeli et al. [17]; Calotă et al. [50]
Internal jugular vein (IJV)	Posterolateral to SP within the carotid sheath	Compression between SP and C1 transverse process (“jugular nutcracker”)	Intracranial hypertension, venous congestion, hemorrhage	Triantafyllou et al. [48]
Facial nerve (CN VII)	Emerges from stylomastoid foramen, lateral to SP base	Variant styloid root morphology alters proximity	Risk of neuropathic pain, nerve traction post-tonsillectomy	Cho et al. [22]; Li et al. [23]

## Data Availability

Please contact the authors for data requests.

## Abbreviations

The following abbreviations are used in this manuscript:

CCA	Common carotid artery
CCAD	Cervical carotid artery dissection
CN	Cranial nerve
CT	Computed tomography
CTA	CT angiography
ECA	External carotid artery
HB	Hyoid bone
ICA	Internal carotid artery
IJV	Internal jugular vein
RC	Reichert’s cartilage
SHC	Stylohyoid complex
SHL	Stylohyoid ligament
SP	Styloid process
US	Ultrasound
